# Editorial: Personalized therapy in ARDS, volume II

**DOI:** 10.3389/fmed.2024.1533593

**Published:** 2024-12-16

**Authors:** Denise Battaglini, Claude E. Guérin, Patricia R. M. Rocco

**Affiliations:** ^1^Department of Surgical Sciences and Integrated Diagnostics, University of Genoa, Genoa, Italy; ^2^Anesthesia and Intensive Care, IRCCS Ospedale Policlinico San Martino, Genoa, Italy; ^3^Division of Intensive Care, Edouard Herriot University Hospital, Lyon, France; ^4^Laboratory of Pulmonary Investigation, Carlos Chagas Filho Institute of Biophysics, Federal University of Rio de Janeiro, Rio de Janeiro, Brazil

**Keywords:** ARDS, mechanical ventilation, ECMO, personalized therapies, biomarkers, sepsis

Acute respiratory distress syndrome (ARDS) can result from a variety of clinical conditions, causing lung injury either directly through local inflammation or indirectly because of systemic inflammatory mediators (1). ARDS is associated with high morbidity and mortality (2). Its pathophysiology is complex, involving the activation and dysregulation of multiple overlapping pathways related to injury, inflammation, and coagulation, both in the lung and systemically (3). Sepsis is the most common cause of ARDS, with pulmonary sepsis (e.g., pneumonia) being the predominant etiology. Non-pulmonary sepsis and non-infectious causes, such as pancreatitis, aspiration of gastric contents, and severe traumatic injuries accompanied by shock and multiple transfusions, also contribute significantly to ARDS cases. Despite decades of experimental research into ARDS biology, these insights have rarely translated into effective therapies (4). This lack of progress is often attributed to the substantial heterogeneity caused by the non-specific clinical definition of ARDS (5). Identifying subphenotypes of ARDS—more homogeneous groups within the broader ARDS population—has emerged as a promising approach to unravel the syndrome's clinical and biological complexity, which many believe is a critical barrier to discovering effective treatments.

Mechanical ventilation (MV) remains the cornerstone of supportive care for ARDS patients. However, improper MV settings can cause complications, most notably ventilator-induced lung injury (VILI) (6). The regional heterogeneity of lung injury, combined with the uneven distribution of mechanical stress during ventilation, results in multiple mechanisms of VILI simultaneously affecting the lungs. While VILI primarily concerns the harmful effects of MV, similar injury pathways can also be triggered by vigorous spontaneous inspiratory efforts, which produce elevated transpulmonary pressures due to highly negative pleural pressures (7).

Furthermore, pro-inflammatory cytokines and chemokines, amplified in the injured lung by harmful MV practices, can enter the systemic circulation, exacerbating systemic inflammation and contributing to non-pulmonary organ failure.

The recognition of ARDS heterogeneity, encompassing its biological, physiological, and radiological aspects, has led to progress in identifying distinct subphenotypes. A precision-medicine approach that effectively classifies ARDS into more homogeneous subphenotypes could enhance our understanding of the syndrome's pathophysiological mechanisms and how these vary across patients, ultimately paving the way for more targeted and effective treatments (8).

This editorial highlights key studies from the Research Topic “*Personalized therapies in ARDS, volume II*,” covering personalized MV techniques, ARDS treatments tailored to etiology, sub-phenotyping, and novel pharmacological strategies.

ARDS, even within a single etiology such as coronavirus disease 2019 (COVID-19), remains a heterogeneous syndrome requiring a personalized MV approach (9). Evidence suggests that lung-protective MV strategies are not adhered to in ~69% of cases (10). This underscores the need for individualized, yet standardized, MV management strategies informed by a comprehensive understanding of the patient's physiology. In this line, Patel et al. conducted a multicenter international randomized clinical trial (RCT) involving 95 ARDS patients to evaluate the feasibility of a physiological model-based decision support system (DSS) for tailoring MV strategies. Patients were randomized to receive either standard care (control) or DSS-guided care (intervention). Although there was no significant difference in average driving pressure between the groups, the intervention arm demonstrated improvements in the oxygenation index and ventilatory ratio during controlled MV mode compared to the control group. Additionally, the DSS led to a significant increase in ventilator adjustments, particularly for pressure settings and respiratory frequency. Importantly, the physiological condition of patients improved when ~60% of DSS recommendations were followed, reinforcing the value of personalized yet repeatable MV strategies. These findings highlight the potential of DSS tools to refine MV management, ultimately improving outcomes in ARDS patients through a more tailored approach.

The equipment shortages during the COVID-19 pandemic presented extreme challenges for many countries. To address the scarcity of devices, alternative strategies were proposed (11, 12). Despite the global resource limitations, the emphasis on personalized MV strategies remained strong, even in scenarios involving shared ventilation between patients. Achanta et al. explored a ventilator-sharing system through benchtop testing that allowed simultaneous ventilation of two patients using a single mechanical ventilator, coupled with a monitoring system to remotely track pulmonary mechanics. The study extended to a swine translational model of lung injury induced by saline lavage to validate the system's functionality in both bench and *in vivo* settings. The benchtop testing demonstrated precise delivery of MV parameters. In the animal model, specific target inspiratory pressures and tidal volumes were set, and the system successfully adjusted for differences between the two pigs. For instance, if one animal reached its target volume or pressure before the other, it experienced a prolonged end-inspiratory pause and stopped receiving flow until both targets were met. While the system introduced a relatively individualized approach to MV settings, addressing some limitations of previous designs, it did not allow for self-tests on the shared ventilator or individualized management of positive end-expiratory pressure (PEEP) (13). These limitations restrict its potential for safe application in human clinical settings.

Over the past few decades, the primary objective of PEEP usage has evolved from solely improving oxygenation to prioritizing lung protection. An “optimal” PEEP simultaneously enhances alveolar recruitment and gas exchange while preventing over distension and minimizing hemodynamic compromise. Once PEEP is set, it is important to regularly reassess its impact on cardiopulmonary function (14, 15). Circulatory failure can result from both too low and too high PEEP levels because they can raise pulmonary vascular resistance, hinder pulmonary circulation, and impede right ventricular performance (16). Xingzheng et al. conducted a prospective paired-design study involving 52 patients with moderate-to-severe ARDS to evaluate the effects of different PEEP levels on cardiovascular function and the incidence of acute cor pulmonale (ACP). The study tested PEEP settings of 5, 10, and 15 cmH_2_O, while maintaining end-inspiratory plateau pressure ≤ 30 cmH_2_O. The incidence of ACP was 25%, with the highest occurrence observed at a PEEP of 15 cmH_2_O. As PEEP levels increased from 5 to 10 and 15 cmH_2_O, the arterial partial pressure of oxygen (PaO_2_)/fraction of inspired oxygen (FiO_2_) ratio improved. However, higher PEEP values were associated with a modest rise in arterial partial pressure of carbon dioxide (PaCO_2_). At a PEEP of 15 cmH_2_O, significant adverse cardiovascular effects were noted, including reduced tricuspid annular plane systolic excursion, increased right ventricular end-diastolic area, elevated pulmonary artery systolic pressure, a 14% reduction in cardiac index, and an 18.1% drop in stroke volume index compared to PEEP settings of 5 or 10 cmH_2_O. Additionally, higher PEEP levels led to a marked decline in the end-diastolic volume index and extravascular lung water index. This study highlights the variability in ACP incidence depending on the PEEP setting in ARDS patients. PEEP levels must be carefully individualized based on the patient's cardiovascular function to optimize outcomes.

Atakul et al. evaluated a closed-loop oxygen control system in 33 pediatric patients (aged 1 month to 18 years) undergoing invasive MV for acute hypoxemic respiratory failure. Patients were randomized to begin with either manual adjustment or a 2-h period of closed-loop oxygen titration. Peripheral oxygen saturation (SpO_2_) levels were maintained within a predetermined target range using either automated or manual FiO_2_ adjustments. The closed-loop oxygen control demonstrated significant advantages: patients managed with the automated system spent a significantly higher percentage of time within the target SpO_2_ range compared to manual adjustment (95.7 vs. 65.6%). Additionally, the automated system achieved a higher median SpO_2_/FiO_2_ ratio (*p* = 0.001), required a lower median FiO_2_ (32.1 vs. 40.6%, *p* = 0.001), and significantly reduced the number of manual adjustments (*p* < 0.001).

Veno-venous extracorporeal membrane oxygenation (VV-ECMO) is employed in patients with severe ARDS to support gas exchange, and deliver safe MV (Pplat), when other strategies fail to yield sufficient benefits (4). Observational studies and meta-analysis found that ARDS patients in VV-ECMO significantly improved outcomes and lowered 60-day mortality more than those on traditional MV (17–19). Current guidelines recommend using VV-ECMO for patients with severe ARDS, as defined by the EOLIA trial eligibility criteria, in centers meeting defined organizational standards. A bibliometric analysis by Lu et al. identified exponential growth in ECMO-ARDS research, involving contributions from 60 countries. The United States and the University of Toronto (Canada) led this research, followed by Germany and China, with Canada, France, and Australia cited most frequently. Recent research has focused on multicenter studies, prone positioning, and COVID-19-related ARDS. ARDS sub-phenotyping based on pulmonary or extra-pulmonary etiology represents another promising approach. Li et al., in a multicenter cohort analysis, compared the sub-phenotypic characteristics of ARDS caused by lung infections vs. non-pulmonary infections. Chronic obstructive pulmonary disease was the most common comorbidity in pulmonary ARDS, while abdominal infections were predominant in non-pulmonary ARDS. Sepsis-induced ARDS was more commonly linked to pulmonary infections caused by *A. baumannii* and *K. pneumoniae*, which were associated with higher mortality rates, worse APACHE II, SOFA, and SAPS II scores, and poorer oxygenation indices compared to ARDS from non-pulmonary infections. Both the PaO_2_/FiO_2_ ratio and ROX index were negatively correlated with prognosis.

The antioxidant effects of vitamin C therapy may mitigate tissue injury induced by oxidative stress in sepsis. However, in adults with sepsis receiving vasopressor therapy in the ICU, those who received intravenous vitamin C had a higher risk of death or persistent organ dysfunction at 28 days than those who received placebo (20). Alissa et al., in their review, highlighted the potential benefits of co-administering vitamin C in patients with sepsis and septic shock caused by pneumonia leading to ARDS.

In conclusion, the presentation of ARDS varies significantly across three key dimensions: (1) etiological, (2) physiological, and (3) biological. This variability emphasizes that a “typical” ARDS does not exist. The lack of a uniform presentation calls for a personalized medicine approach. Advanced sub-phenotyping and integrative analyses of these variations may uncover additional treatable traits, paving the way for more targeted therapies.
